# Identification of Thyroid Hormones and Functional Characterization of Thyroid Hormone Receptor in the Pacific Oyster *Crassostrea gigas* Provide Insight into Evolution of the Thyroid Hormone System

**DOI:** 10.1371/journal.pone.0144991

**Published:** 2015-12-28

**Authors:** Wen Huang, Fei Xu, Tao Qu, Rui Zhang, Li Li, Huayong Que, Guofan Zhang

**Affiliations:** 1 Key Laboratory of Experimental Marine Biology, Institute of Oceanology, Chinese Academy of Sciences, Qingdao, China; 2 University of Chinese Academy of Sciences, Beijing, China; 3 Laboratory for Marine Biology and Biotechnology, Qingdao National Laboratory for Marine Science and Technology, Qingdao, China; 4 National & Local Joint Engineering Laboratory of Ecological Mariculture, Institute of Oceanology, Chinese Academy of Sciences, Qingdao, China; Laboratoire de Biologie du Développement de Villefranche-sur-Mer, FRANCE

## Abstract

Thyroid hormones (THs) play important roles in development, metamorphosis, and metabolism in vertebrates. During the past century, TH functions were regarded as a synapomorphy of vertebrates. More recently, accumulating evidence has gradually convinced us that TH functions also occur in invertebrate chordates. To date, however, TH-related studies in non-chordate invertebrates have been limited. In this study, THs were qualitatively detected by two reliable methods (HPLC and LC/MS) in a well-studied molluscan species, the Pacific oyster *Crassostrea gigas*. Quantitative measurement of THs during the development of *C*. *gigas* showed high TH contents during embryogenesis and that oyster embryos may synthesize THs endogenously. As a first step in elucidating the TH signaling cascade, an ortholog of vertebrate TH receptor (TR), the most critical gene mediating TH effects, was cloned in *C*. *gigas*. The sequence of *CgTR* has conserved DNA-binding and ligand-binding domains that normally characterize these receptors. Experimental results demonstrated that *CgTR* can repress gene expression through binding to promoters of target genes and can interact with oyster retinoid X receptor. Moreover, *CgTR* mRNA expression was activated by T4 and the transcriptional activity of *CgTR* promoter was repressed by unliganded *CgTR* protein. An atypical thyroid hormone response element (CgDR5) was found in the promoter of *CgTR*, which was verified by electrophoretic mobility shift assay (EMSA). These results indicated that some of the *CgTR* function is conserved. However, the EMSA assay showed that DNA binding specificity of *CgTR* was different from that of the vertebrate TR and experiments with two dual-luciferase reporter systems indicated that l-thyroxine, 3,3′,5-triiodothyronine, and triiodothyroacetic acid failed to activate the transcriptional activity of *CgTR*. This is the first study to functionally characterize TR in mollusks. The presence of THs and the functions of *CgTR* in mollusks contribute to better understanding of the evolution of the TH system.

## Introduction

Thyroid hormones (THs), l-thyroxine (T4) and 3,3′,5-triiodothyronine (T3), play important roles in the development, growth, metamorphosis, and metabolism of vertebrates [1–3]. These TH functions have been extensively explored in a wide range of vertebrate species; for example a strong evidence for TH effects in the development is that amphibian metamorphosis is obligatorily initiated and sustained by THs [4]. The TH signaling pathway has also been well studied [5]. THs (mainly the pro-hormone T4) are synthesized by thyroid peroxidase in the thyroid gland using iodine and tyrosine. These THs are then transported to peripheral tissues and transformed into the active form T3 by iodothyronine deiodinases, which can also inactivate THs. THs exert their effects, for the most part, through binding to the nuclear receptor, the thyroid hormone receptor (TR), to mediate transcriptional regulation of target genes. TR is a ligand-regulatable receptor that represses the transcription of a target gene in the absence of T3 and activates the transcription in the presence of T3, in most cases. TRs bind to thyroid hormone response elements (TREs) within the promoters of the target genes. The typical TRE comprises two direct repeats of the half-site (AGGTCA) separated by 0 or 4 base pairs (commonly named DR0 or DR4). Vertebrate TR binds to DR4 as a heterodimer with the retinoid X receptor (RXR), another member of the nuclear receptor family [6].

In contrast to the extensively studied THs in vertebrates, the TH-related studies in invertebrates are very limited. Consequently, during the last century, THs were regarded as a synapomorphy of vertebrates [1]. More recently, accumulating evidence from physiological and molecular studies has gradually convinced us that TH-related functions and signaling pathways are also present in the invertebrate chordates Cephalochordata and Urochordata. THs have been detected in amphioxus and ascidians, and the endostyle of these organisms is widely considered to be a homolog of the vertebrate thyroid gland [7,8]. T4, T3, and triiodothyroacetic acid (TRIAC, a TH derivative) can induce the metamorphosis of amphioxus, but only TRIAC was bound by amphioxus TR [9]. Most of the genes involved in TH signaling in vertebrates have also been found in both amphioxus and ascidians [10,11]. Thus, the origin of TH functions dates back at least to invertebrate chordates.

Nevertheless, TH-related studies in non-chordate invertebrates are limited to and scattered among several species. In echinoderms, metamorphosis of the sea urchin *Lytechinus variegatus* was reported to be accelerated by T4, and the larvae seem to accumulate THs from algae and synthesize THs endogenously [12]. In mollusks, metamorphosis is induced by T4 and T3 in two abalone species (*Haliotis discus* and *H*. *gigantea*) [13]. A deiodinase homolog was cloned and THs were detected in the hemolymph of the scallop *Chlamys farreri* by enzyme-linked immunosorbent assay (ELISA) [14]. In the Platyhelminthes, three TR homologs were cloned in *Schistosoma mansoni* and *S*. *japonicum* [15,16]. However, this sporadic evidence was far from sufficient to convince the scientists to change their opinion that THs are restricted to chordates. On the basis of these previous studies, it is reasonable to assume that THs are also functional in non-chordate invertebrates and that the origin and evolution of THs dates back to the common ancestor of protostomes and deuterostomes.

In this context, a study was conducted to identify THs and functionally characterize TR during the development of the Pacific oyster *Crassostrea gigas* (Thunberg), a well-studied molluscan species owing to its importance in aquaculture and evolutionary studies. THs were qualitatively and quantitatively detected during the development of *C*. *gigas*. The single ortholog of the vertebrate TR, *CgTR*, was cloned and its protein expression profile was examined. The functional characteristics of CgTR were studied using various molecular approaches: yeast two-hybrid assay, dual-luciferase reporter assay, and electrophoretic mobility shift assay (EMSA).

## Materials and Methods

### Oyster sample collection and TH extraction

The Pacific oyster larvae were sampled at representative developmental stages from a cultured population in Qingdao, China. Because of a relatively long time to through this developmental stage, pediveliger larvae were selected for treatment with T4. Larvae were cultured in filtered sea water containing 1.29 × 10^−8^ M T4 and sampled at 1, 3, 6, and 12 h post-treatment. Samples were initially frozen in liquid nitrogen and then stored at -80°C. Oyster larval samples for TH measurement were stored for no more than 2 months. As oyster larvae are minute (60–380 μm), an enormous number (each sample >10,000 individuals) of larvae were used to facilitate the quantitative measurement of THs throughout the developmental stages. Samples were processed as previously described [12] with some modifications. In brief, material was ground in liquid nitrogen and completely dried in a vacuum freeze drier (Biocool, Beijing, China). Approximately 0.2 g of dried samples was then weighed and 0.01 M NaOH was added in a ratio of 5 mL/g. Extraction was performed at 4°C overnight. After centrifugation at 8,000 rpm for 5 min at 4°C, the liquid supernatants were subjected to qualitative and quantitative measurements of THs.

### High-performance liquid chromatography (HPLC) analysis

HPLC was performed as previously reported [17]. In brief, standard T3 and T4 (Sigma Aldrich, St. Louis, MO, USA) were dissolved in 0.01 M NaOH and mixed in the ratio 1:1 (v/v) with the eluent. A YMC-Pack ODS-AQ analytical column (Waters, Milford, MA, USA) was used and the eluent was a mixture of CH_3_OH-H_2_O with 2% acetic acid, at a volume ratio 65:35. The flow rate was 1 mL/min and detection was performed with a variable-wavelength UV-visible detector at 240 nm.

### Liquid chromatography with tandem mass spectrometry (LC/MS)

Standard T3, T4 (Sigma Aldrich, USA), and trochophore samples were dissolved in methanol. The standard solutions and sample extractions were analyzed by LC/MS by positive ion electrospray ionization. Separation was achieved using a Nexera X2 LC-30AD liquid chromatography system (SHIMADZU, Kyoto, Japan) with water and methanol as the eluents (each contained 0.025% formic acid and 1 mM NH_4_Ac). A 10-μL sample volume was injected into an XR-ODS column (SHIMADZU, Japan) and then separated using an eluent gradient as follows: 80% aqueous eluent for 1 min, 35% aqueous eluent for 2 min, 80% aqueous eluent for 2 min at a 0.3 mL/min flow rate. A SCIEX Triple Quad 4500 mass spectrometer (Applied Biosystems, Foster City, CA, USA) was employed for mass spectrometry detection of multiple reaction monitoring ions 777.6 (Q1)/731.6 (Q3), 777.6 (Q1)/760.8 (Q3), and 777.6 (Q1)/633.9 (Q3) for T4 and 651.6 (Q1)/605.8 (Q3), 651.6 (Q1)/508.1 (Q3), and 651.6 (Q1)/634.8 (Q3) for T3.

### Quantitative measurement of THs in oyster larvae

The above TH extractions were quantitatively tested using Elecsys T3 and Elecsys T4 kits (Roche Diagnostics, Mannheim, Germany) on an Elecsys 2010 automated electrochemical immuno-analyzer (Roche Diagnostics, Germany) following the manufacturer’s instructions [18]. TH contents were determined automatically according to the standard curve. A negative control (0.01 M NaOH) was tested in each batch and the results were used for calibration. The detection limits of this system were 5.40 nmol/L for T4 and 0.30 nmol/L for T3 and the lowest value detected were 9.30 nmol/L for T4 and 0.62 nmol/L for T3, respectively. Thus, all detected values were located in convincing range. The recovery of our processing system was assessed by pike in standard T4 and T3 in larval samples before the samples were ground in liquid nitrogen. The mean recovery of standard T4 and T3 was 57.65% ± 1.31% and 27.22% ± 1.68%, respectively. For protein analysis, approximately 0.01 g of dried samples was dissolved for 1 h in 1 mL cell lysis buffer RIPA (Solarbio, Beijing, China) at room temperature, and protein assay was conducted using a BCA kit (Solarbio, China) following the manufacturer’s instructions. The larval content of THs was corrected using the mean recovery values and expressed as microgram THs per gram protein (μg/g protein).

### Genes and promoter cloning

Gene cloning was conducted as previously described [19]. Briefly, on the basis of predicted coding sequences (CDS), primers were designed to amplify the middle fragments. Rapid amplification of cDNA 3′ and 5′ end primers were then designed to obtain the full-length cDNA. The full-length cDNA was located on the Pacific oyster genome, and we extracted approximately 3 kb of genomic DNA from the 5′ upstream region as a reference to design cloning forward primers. Reverse primers were designed based on the 5′-UTR sequence.

### Sequence analysis and phylogenetic tree construction

Sequence analysis was conducted using known invertebrate TRs and representative vertebrate TRs. The DNA-binding domain (DBD) and ligand-binding domain (LBD) sequences of TRs, determined using the Pfam web services (http://pfam.xfam.org/) [20], were extracted and used to construct phylogenetic trees. Multiple alignments were performed with ClustalW [21] and phylogenetic trees were constructed using four different algorithms (i.e., the maximum likelihood, maximum parsimony, neighbor-joining, and minimum-evolution) in MEGA5.0 software (http://www.megasoftware.net) [22]. Support values of the phylogenetic trees were derived by bootstrapping with 1000 replicates.

### RNA isolation, cDNA synthesis, and quantitative real-time PCR

RNA isolation and cDNA synthesis experiments were conducted using a TRIzol reagent (Invitrogen, Carlsbad, CA, USA) and a PrimeScript RT reagent Kit (TaKaRa, Shiga, Japan), respectively, according to manufacturers’ instructions. Quantitative real-time PCR (qRT-PCR) was conducted as previously described [19]. The ribosomal protein S18 (*RS18)* gene was used as an internal control [23] and the 2^−ΔΔCt^ method was used to calculate the expression level of target genes [24].

### Western blotting

For western blotting, a custom-made polyclonal antibody against CgTR (anti-CgTR) was prepared by Abmart, Inc. (Shanghai, China). A peptide (KRKLIEENREKR) was selected and produced by chemosynthesis for polyclonal antibody production. Oyster larvae in representative stages were ground in liquid nitrogen and dissolved in RIPA. Total proteins (~50 μg) from each sample were separated on a 12% polyacrylamide gel using SDS-PAGE and transferred onto a PVDF membrane. The membranes were blocked in 5% skimmed milk for 1 h and incubated with polyclonal antibodies at 4°C overnight. After washing three times (5 min each) in Tris-buffered saline, the membranes were incubated with horseradish peroxidase-conjugated goat anti-rabbit IgG (Roche Diagnostics, Germany) for 1 h. Finally, the membranes were washed three additional times, and the bands on the membranes were visualized using Western Lightning Plus-ECL (Perkin Elmer, Waltham, MA, USA). Rabbit anti-β-actin IgG (ABclonal Technology, Cambridge, MA, USA) was used as a reference.

### Yeast two-hybrid assay

Yeast two-hybrid assay was conducted using the Matchmaker^™^ Gold Yeast Two-Hybrid System (Clontech, Palo Alto, CA, USA) according to the manufacturer’s instructions. Full-length cDNAs encoding CgTR and CgRXR were inserted into pGAD-T7 and pGBK-T7 vectors to create pGAD-CgTR and pGBK-CgRXR, respectively. Y187 and Y2HGold yeast strains were transfected with 1 μg of pGAD-CgTR and pGBK-CgRXR plasmids, respectively. The following cotransformations were performed: pGAD-CgTR/pGBK-CgRXR, pGAD-T7(empty)/pGBK-CgRXR, and pGAD-CgTR/pGBK-T7(empty). The cotransformed yeasts were spread on SD⁄–Trp⁄–Leu medium and SD/–Ade/–His/–Leu/–Trp medium plus X-α-Gal/AbA.

### Recombinant protein expression

Recombinant protein expression experiments were performed using the prokaryotic expression vector pET30a (Novagen, George Town, KY, USA) in *Escherichia coli* BL21(DE3) (TransGen Biotech, Beijing, China). Briefly, cDNAs coding full-length CgTR (designated as rCgTR) and truncated CgTR (containing the variable A/B domain and the conserved DBD domain, i.e., from amino acid 1 to 240, designated as rCgTR1-240) were inserted into the pET30a plasmid. Target plasmid-transfected *E*. *coli* grown on kanamycin (50 μg/mL) were added to liquid broth and grown to OD_600nm_ = 0.6. A final concentration of 0.5 mM isopropyl β-D-1-thiogalactopyranoside (IPTG) was then added to the culture, and *E*. *coli* were incubated for an additional 12 h to express the soluble protein. Finally, *E*. *coli* were subjected to ultrasonication and the soluble bacterial protein extracts were stored at -80°C for subsequent analysis. Recombinant proteins were identified by SDS-PAGE on 12% gels stained with Coomassie Brilliant Blue R250.

### Electrophoretic mobility shift assay (EMSA)

EMSA was performed using a LightShift Chemiluminescent EMSA kit (Pierce Biotechnology, Rockford, IL, USA) according to the manufacturer’s instructions. Briefly, the proteins were produced using a prokaryotic expression system (see above). The following single-stranded oligonucleotides containing the consensus half-site AGGTCA were synthesized as previously reported [25] and labeled with biotin: DR0: 5′-CCGTAAGGTCAAGGTCACTCG-3′; DR1: 5′-CCGTAAGGTCACAGGTCACTCG-3′; DR2: 5′-CCGTAAGGTCACAAGGTCACTCG-3′; DR3: 5′-CCGTAAGGTCACAGAGGTCACTCG-3′; DR4: 5′-CCGTAAGGTCACAGGAGGTCACTCG-3′; DR5: 5′-CCGTAAGGTCACCAGGAGGTCACTCG-3′. A putative TRE corresponding to the CgTR promoter region from -1043 to -1070 was also synthesized: CgDR5: 5′-GTCCGTGATCACGGGTAGGTCAAACTG-3′. Each oligonucleotide was annealed with its complementary strand. In order to compare the affinities of CgTR protein to DR0-DR5, the same concentrations of DR0-DR5 and CgTR protein were used in binding reactions. Cold specific competitions were performed using 100-fold concentrations of cold probe (unlabeled oligonucleotides same as the labeled ones). Supershift experiments were performed in the presence of a monoclonal anti-His antibody (TIANGEN Biotech, Beijing, China).

### Dual-luciferase reporter assay

Two types of dual-luciferase reporter assay were applied in this study. First, the expression vector pcDNA3.1 (Invitrogen, USA) and reporter vector pGL3-Basic (Promega, Madison, WI, USA) were used in the dual-luciferase reporter system with pcDNA3.1/pGL3. The full-length CDSs of CgTR were subcloned into pcDNA3.1 (pcDNA3.1-CgTR) and the cloned *CgTR* promoter (nucleotide -3047 to -1) was fused with pGL3-Basic (pGL3-CgTRpromoter). We also employed a dual-luciferase reporter system with pGal4/pUAS, using the plasmids pGal4-hsTRβ(LBD) and pUAS-luc (kindly provided by Professor Xinru Wang). The LBD of CgTR was linked behind the DBD of Gal4 in plasmid pGal4-CgTR(LBD). The Gal4-responsive luciferase reporter plasmid pUAS-luc contained four copies of the Gal4-binding site (UAS). Dual-luciferase reporter assays were performed using HEK293T cells (ATCC, Manassas, VA, USA) seeded into 96-well plates and cultured in Dulbecco's Modified Eagle Media (Invitrogen, USA) supplemented with 10% fetal bovine serum, penicillin (100 U/mL), and streptomycin (100 U/mL). After 24 h, each well was transfected with 20 ng reporter vector, 5 ng pRL-CMV (Promega, USA), and 0, 20, 40, or 80 ng pcDNA3.1-CgTR. The total amount of DNA in each well was equalized with pcDNA3.1. At 24 h post-transfection, luciferase activity was measured using the Dual-Luciferase Reporter Assay System (Promega, USA) in a Varioskan Flash multimode reader (Thermo Scientific, Waltham, MA, USA). For the dual-luciferase reporter assays performed in the presence of different concentrations of THs, each well was transfected with 20 ng reporter vector, 5 ng pRL-CMV (Promega, USA), and 20 ng expression plasmid. T4, T3, and TRIAC were each dissolved in 0.01 M NaOH in a concentration series and added to the wells 4–6 h after transfection to obtain final concentrations ranging from 10^−12^ to 10^−6^ M.

## Results

### Qualitative and quantitative measurements of THs in the development of the Pacific oyster

**Fig 1:**
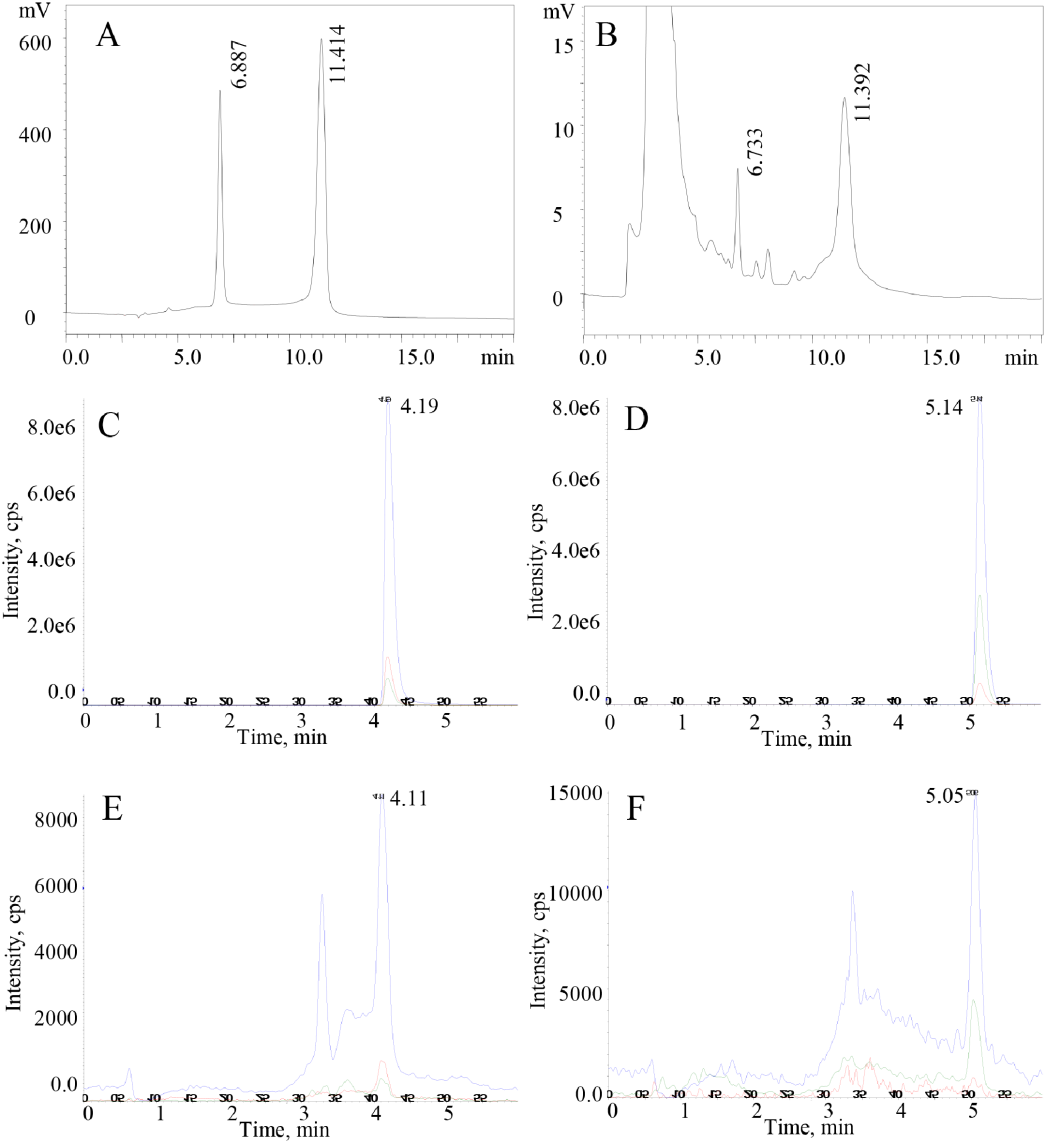
Qualitative measurement of T3 and T4 by HPLC (A, B) and LC/MS (C, D, E, F). In the HPLC assay, standard T3 and T4 were separated at 6.887 min and 11.414 min (A), respectively. Extracts from the blastula contain T3 (6.733 min) and T4 (11.392 min) (B). In LC/MS, standard T3 and T4 were separated at 4.19 min (C) and 5.14 min (D), respectively. Extracts from the trochophore contain T3 (4.11 min, E) and T4 (5.05 min, F). The blue, red and green line in Fig 1C and E represent the intensity value detected by mass spectrometry of multiple reaction monitoring ions 651.6 (Q1)/605.8 (Q3), 651.6 (Q1)/508.1 (Q3), and 651.6 (Q1)/634.8 (Q3) for T3, respectively. The blue, green and red line in Fig 1C and E represent that of multiple reaction monitoring ions 777.6 (Q1)/731.6 (Q3), 777.6 (Q1)/760.8 (Q3), and 777.6 (Q1)/633.9 (Q3) for T4, respectively. Experimental conditions are described in the text.

The components of standard T3 and T4 were detected by HPLC using a UV-visible detector (Fig 1A and 1B) and subsequently confirmed by liquid chromatography with tandem mass spectrometry (LC/MS) in oyster blastula and trochophore extracts, respectively (Fig 1C–1F). These results indicated that oyster larvae contain T3 and T4.

**Fig 2:**
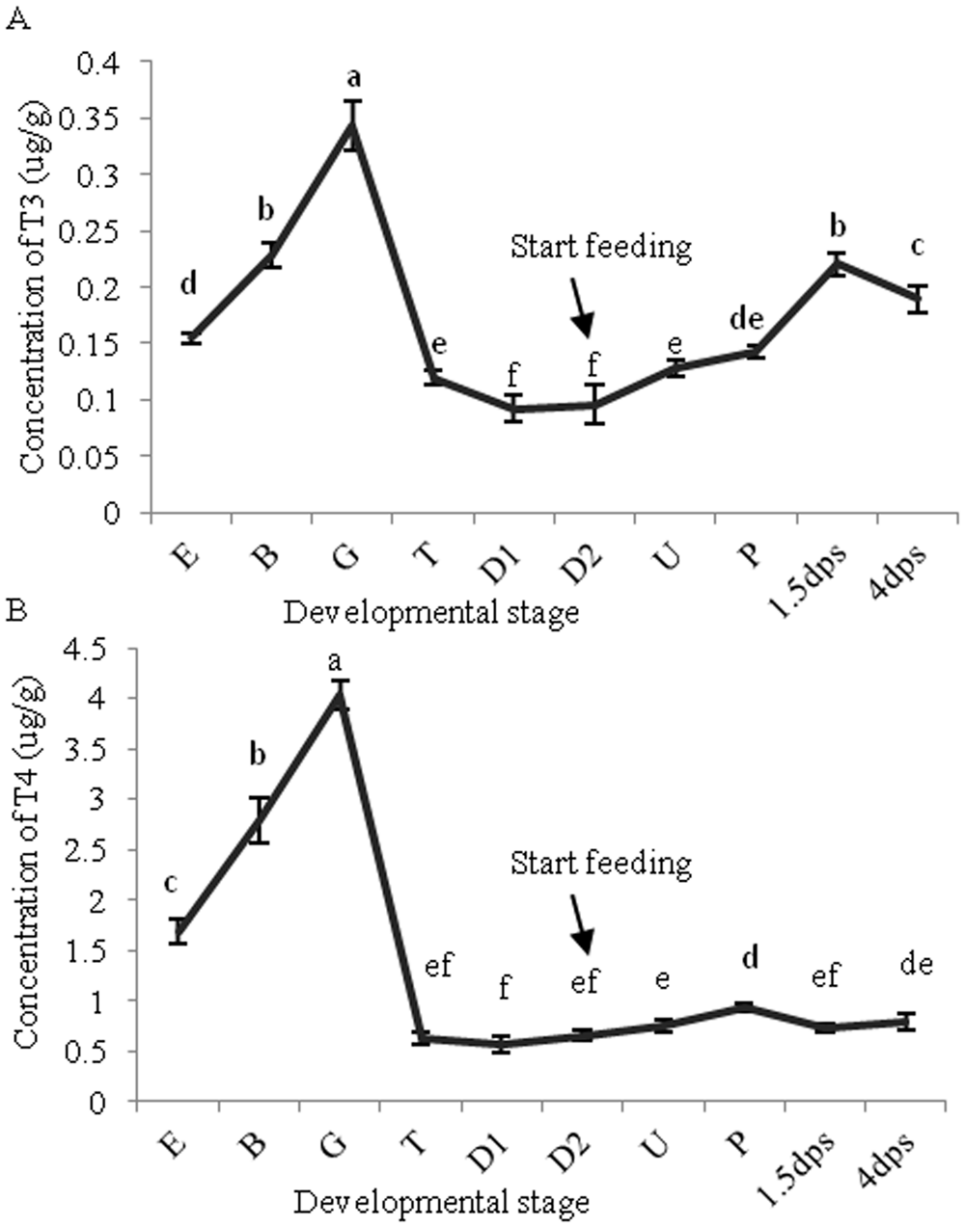
T3 (A) and T4 (B) content (μg/g protein) in the developmental stages of *C*. *gigas*. Larvae started feeding on *Isochrysis galbana* at D2 stage. Data are presented as the mean ± SD of triplicate independent experiments and were analyzed by one-way ANOVA in SPSS. TH contents at each time point that are not significantly different from one another have been marked with the same letter (*P* ≥ 0.05). E: egg, B: blastula, G: gastrula, T: trochophore, D: D-shape, U: umbo, P: pediveliger, dps, days post-settlement.

The variation in TH contents could provide clues as to their possible role in different developmental stages. The TH contents were measured accordingly during oyster developmental stages from unfertilized eggs to 4 days post-settlement (dps). To investigate whether TH contents increased after feeding on algae and in metamorphosis progress respectively, additional stages D2 and 1.5 dps were added in quantitative measure of THs, next to the representative developmental stages. THs were detected throughout the examined developmental stages (Fig 2A and 2B). An obvious wave of THs was discerned during embryogenesis from the egg (E) to the trochophore (T). In the ensuing developmental stages, T3 content gradually increased, whereas T4 content remained at a low level from the D-shape larva (D1) stage to 4 dps.

### Gene cloning and sequence analysis

An oyster TR (*CgTR*) was found in a previous study using bioinformatics methods [26]. To study the molecular mechanism of TH effects, the full-length cDNA of CgTR was cloned and deposited in GenBank (accession number KP271450). The cDNA of *CgTR* contained 3,003 nucleotides and the CDS contained 1,440 nucleotides, coding for a protein of 479 amino acids. Alignment of the amino acid sequences with known TR orthologs showed that the sequence of CgTR was highly conserved (shown in S1 Fig). The most conserved domains are the DBD and the LBD, with 74–78% and 52–59% identity, respectively, to chordate TRs. The 3047-kb promoter of *CgTR* was also cloned and was used in the dual-luciferase reporter system with pcDNA3.1/pGL3. A putative atypical TRE (CgDR5) was found in the *CgTR* promoter at -1043 to -1070, with five base pairs inserted between the direct repeats and two mismatches in the first half-site, compared with the consensus AGGTCA (S2 Fig).

**Fig 3:**
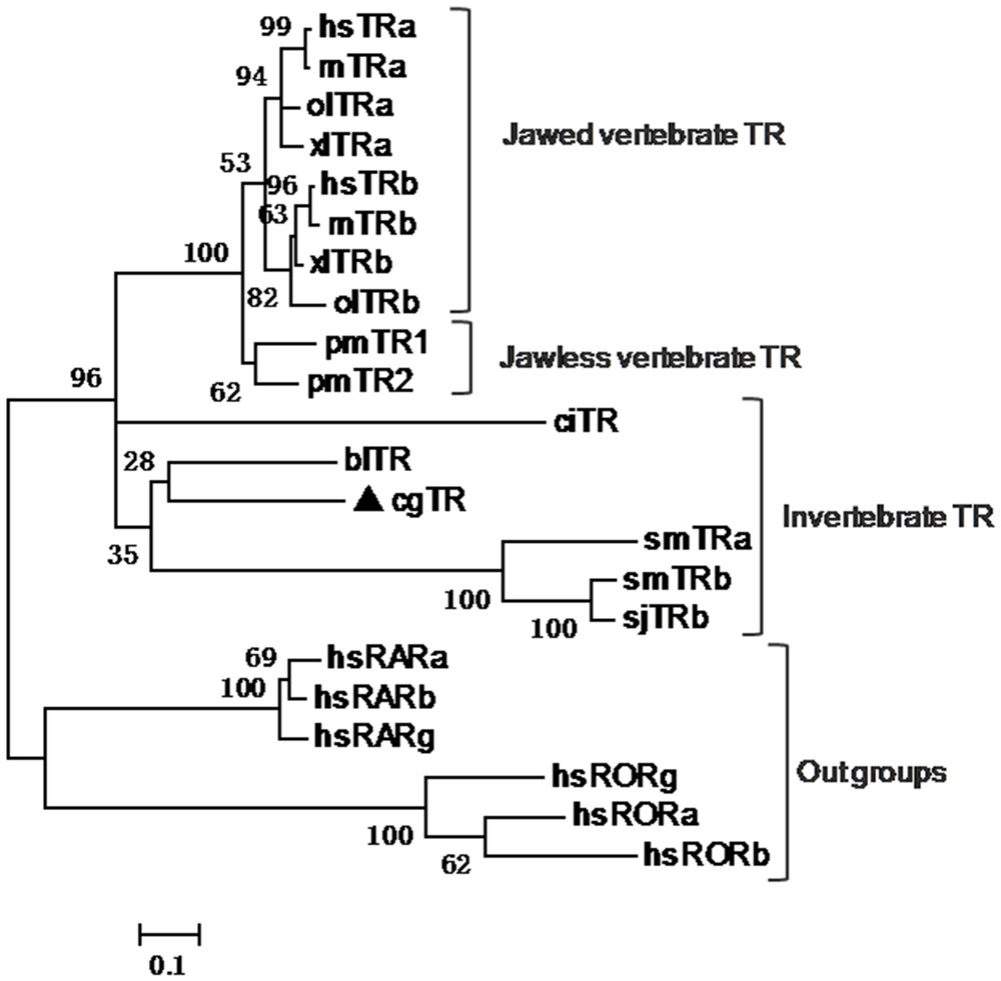
Phylogenetic analysis of TR. The phylogenetic tree was derived from amino sequences of the DNA-binding domain plus ligand-binding domain of TRs. CgTR is marked with a black triangle. The human RARs and RORs were used as out groups. Protein domains were predicted by Pfam. The phylogenetic tree was constructed using the Maximum Likelihood algorithm using MEGA5.0 software. TR, thyroid hormone receptor; RAR, retinoid acid receptor; ROR, RAR-related orphan receptors; hs, *Homo sapiens* (hsTRa, AB307686; hsTRb, M26747; hsRARg, M24857; hsRORa, U04897; hsRORg, U16997; hsRARa, X06614; hsRARb, X07282; hsRORb, Y08639); rn, *Rattus norvegicus* (rnTRa, M18028; rnTRb, J03933); xl, *Xenopus laevis* (xlTRa, M35343; xlTRb, M35360); ol, *Oryzias latipes* (olTRa, AB114860; olTRb, AB114861); pm, *Petromyzon marinus* (pmTR1, DQ320317; pmTR2, DQ320318); bl, *Branchiostoma lanceolatum* (blTR, EF672345); ci, *Ciona intestinalis* (ciTR, NM_001032486); sm, *Schistosoma mansoni* (smTRa, AY395038; smTRb, AY395039); sj, *Schistosoma japonicum* (sjTRb, JX111998); cg, *Crassostrea gigas* (cgTR, KP271450).

Phylogenetic trees were derived from the amino acid sequences of the DBD plus LBD of TRs to investigate the evolutionary relationship of these TRs. Four phylogenetic trees constructed using four different algorithms were congruent. We show the most supported tree topology inferred from the maximum likelihood analysis (Fig 3). In this phylogenetic tree, the TRs from invertebrate and vertebrates were grouped together with high bootstrap value support. The jawless vertebrate TRs (pmTR1 and pmTR2) clustered together and were distant from the jawed vertebrate TRs. In the invertebrate group, the Schistosoma TRs were clustered, and the relationship of remaining invertebrate TRs, *Branchiostoma lanceolatum* TR, *Ciona intestinalis* TR, and *C*. *gigas* TR were unresolved. The evolution of invertebrate TRs is currently unclear, due to the limited number of cloned invertebrate TRs.

### Expression analysis of *CgTR* in developmental stages and in T4 treatment

**Fig 4:**
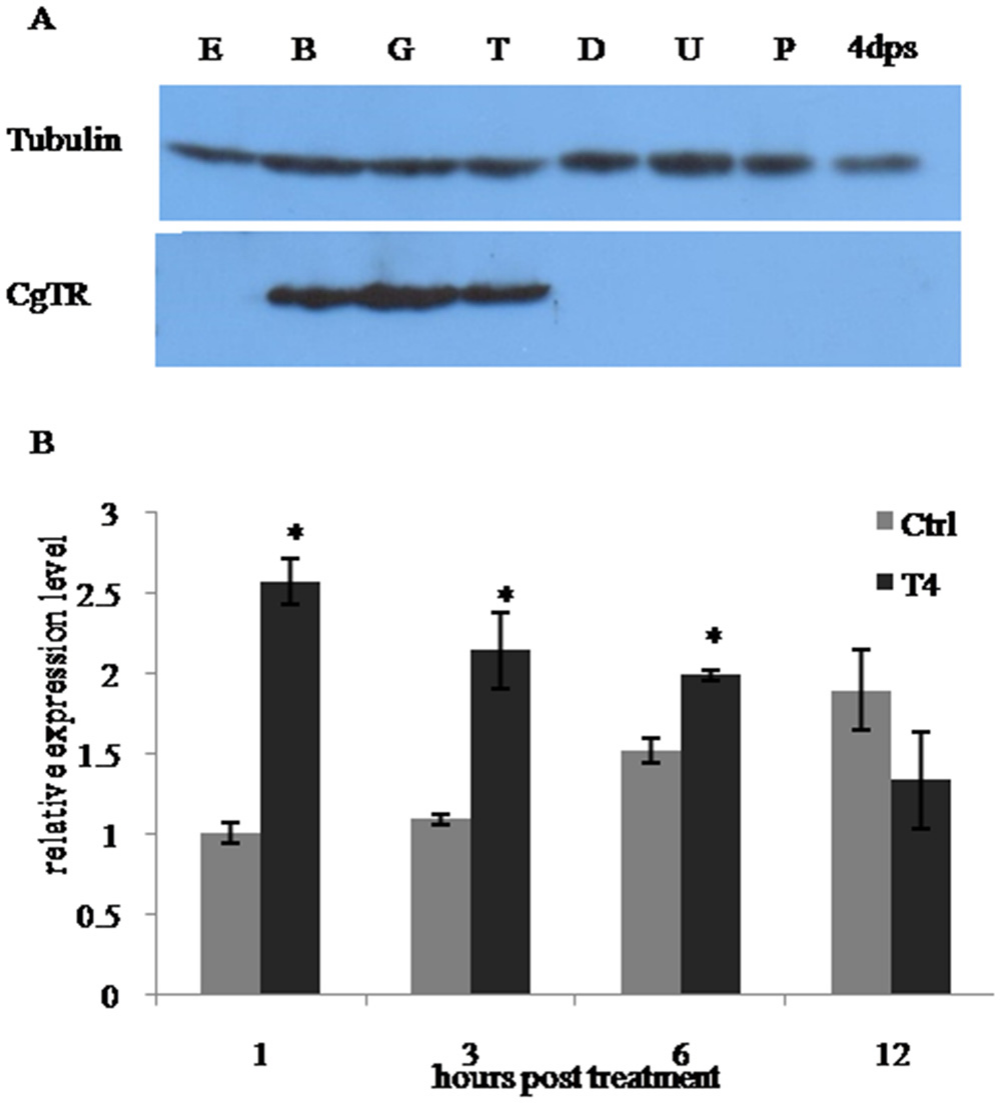
Expression analysis of *CgTR* in developmental stages and in T4 treatment. (A) Western blot analysis of CgTR protein expression in oyster developmental stages. Tubulin was used as an internal reference. (B) Temporal expression of *CgTR* mRNA detected by qRT-PCR during T4 treatment. *RS18* gene expression was used as an internal control. Data are normalized to the control group at 1 h and displayed as the mean ± SD of triplicate independent experiments.

The binding of the custom-made polyclonal antibody to CgTR was first confirmed by the western blot assay (S3 Fig). The western blot was performed to determine the protein expression patterns of CgTR in representative developmental stages. The results (Fig 4A) showed that the CgTR protein was detected in the blastula (B), gastrula (G), and trochophore (T) stages, but it was undetectable in other stages using this method. The most abundant CgTR protein was observed in the G stage. This expression pattern was consistent with the variation tendency of T4 and T3 contents, indicating that CgTR is involved in THs signaling. Due to the limited total amount of each sample collected in the T4 treatment, qRT-PCR was performed to investigate the influence of exogenous T4 in *CgTR* mRNA expression *in vivo*. The results showed that *CgTR* mRNA expression level was induced significantly at 1, 3, and 6 h post-treatment, but not at 12 h post-treatment (Fig 4B). This result indicates that THs could induce the expression of *CgTR in vivo*.

### *CgTR* interacts with *CgRXR*

**Fig 5:**
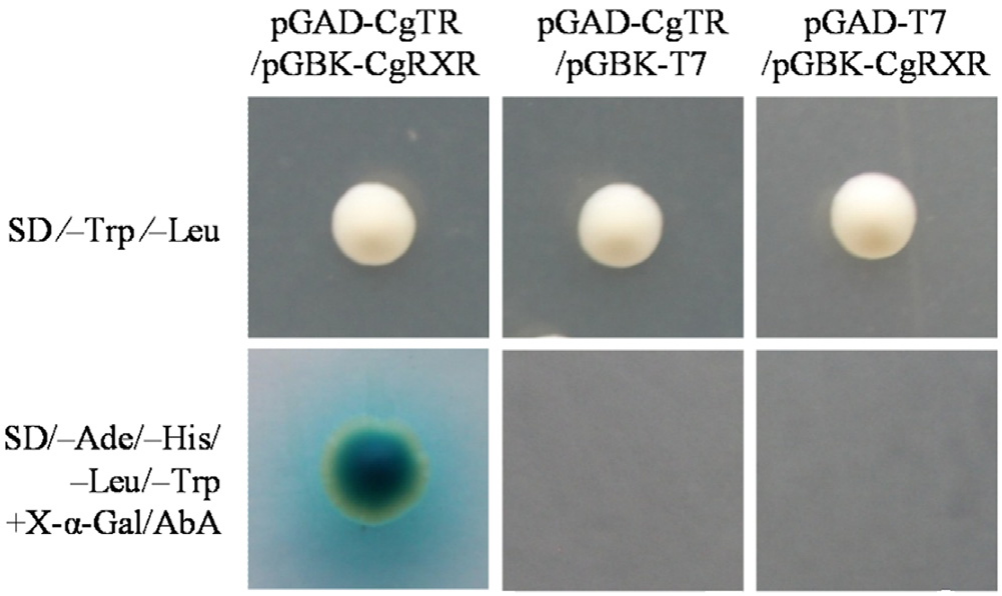
Yeast two-hybrid assay showing the interaction between *CgTR* and *CgRXR*. Cotransformants containing pGAD-CgTR/pGBK-CgRXR, pGAD-T7(empty)/pGBK-CgRXR, and pGAD-CgTR/pGBK-T7(empty) grew on the SD⁄–Trp⁄–Leu medium, but only pGAD-CgTR/pGBK-CgRXR grew on the SD/–Ade/–His/–Leu/–Trp medium plus X-α-Gal/AbA

A yeast two-hybrid assay was performed to investigate whether CgTR interacts with CgRXR. The results showed that pGAD-CgTR/pGBK-CgRXR, pGAD-T7(empty)/pGBK-CgRXR, and pGAD-CgTR/pGBK-T7(empty) grew on the SD⁄–Trp⁄–Leu medium, but only pGAD-CgTR/pGBK-CgRXR grew on the SD/–Ade/–His/–Leu/–Trp medium plus X-α-Gal/AbA (Fig 5). The results demonstrated that pGAD-CgTR and pGBK-CgRXR cannot independently activate a reporter gene and verified the interaction between CgTR and CgRXR.

### DNA binding specificity of *CgTR* is different from vertebrate TRs

**Fig 6:**
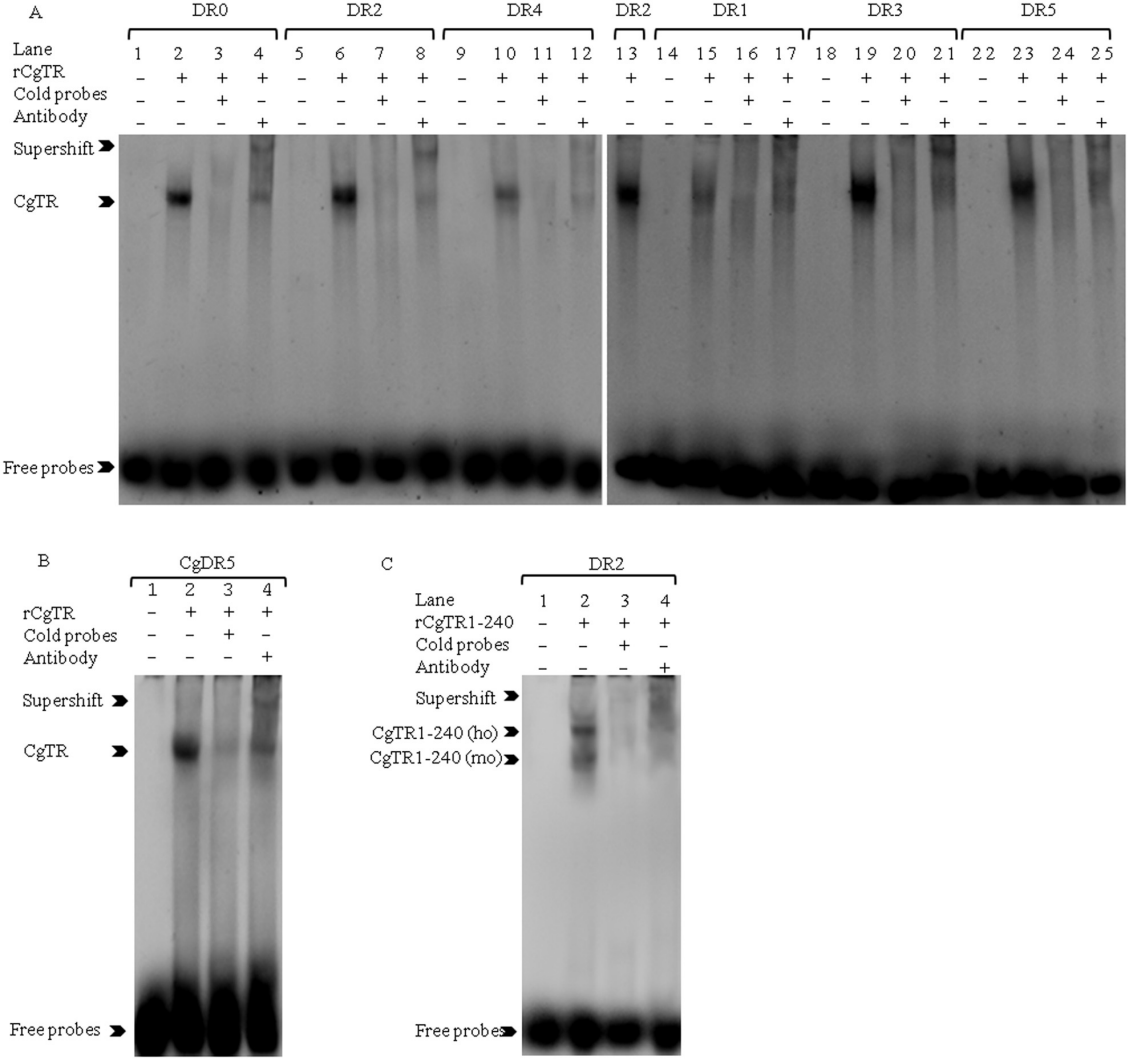
EMSA shows the DNA binding properties of *CgTR in vitro*. (A) Recombinant CgTR (rCgTR) bound to probes DR0-DR5. Lanes 1, 5, 9, 14, 18, and 22 contain the reaction mixture without rCgTR as negative controls; Lanes 2, 6, 10, 15, 19, and 23 contain rCgTR; Lanes 3, 7, 11, 16, 20, and 24 contain rCgTR with a 100 fold concentration of cold probe; Lanes 4, 8, 12, 17, 21, and 25 contain rCgTR with antibody. Lane 13 contains rCgTR as an internal reference in the second plate to compare the binding intensity of DR0-DR5. As only one band was discerned, it is difficult to determine whether the bands are a homodimer or monomer. (B) EMSA demonstrated that CgTR can bind to the CgDR5. Lane 1 contains the reaction mixture without rCgTR as a negative control; Lane 2 contain rCgTR; Lane 3 contains rCgTR with a 100 fold concentration of cold probe; Lane 4 contains rCgTR with antibody. (C) Recombinant CgTR containing amino acids from 1 to 240 (rCgTR1-240) can bind to DR2 as a homodimer (ho) and monomer (mo). Lane 1 contains the reaction mixture without rCgTR1-240 as a negative control; Lane 2 contains rCgTR1-240; Lane 3 contains rCgTR1-240 with a 100 fold concentration of cold probe; Lane 4 contains rCgTR1-240 with antibody.

EMSAs were conducted to determine the DNA binding specificity of CgTR. Seven probes containing a direct repeat of the half-site spaced with 0–5 base pairs (DR0-DR5) and a putative TRE (CgDR5) found in the promoters of *CgTR* were employed. A gel shift was observed when biotin-labeled probes were added to the recombinant protein rCgTR. rCgTR was bound to DR0-DR5 (Fig 6A) and CgDR5 (Fig 6B). The order of rCgTR binding intensity to DR0-DR5 was DR3 > DR2 > DR5> DR0 > DR4 > DR1. To examine whether the DBD domain could bind to the probes *in vitro*, an EMSA was performed with DR2 as the probe and rCgTR1-240 as the target protein. A gel shift caused by rCgTR1-240 demonstrated that the ability to bind to promoters was preserved by the DBD domain (Fig 6C).

### Transcriptional activity of *CgTR* was not stimulated by T4, T3, or TRIAC

**Fig 7:**
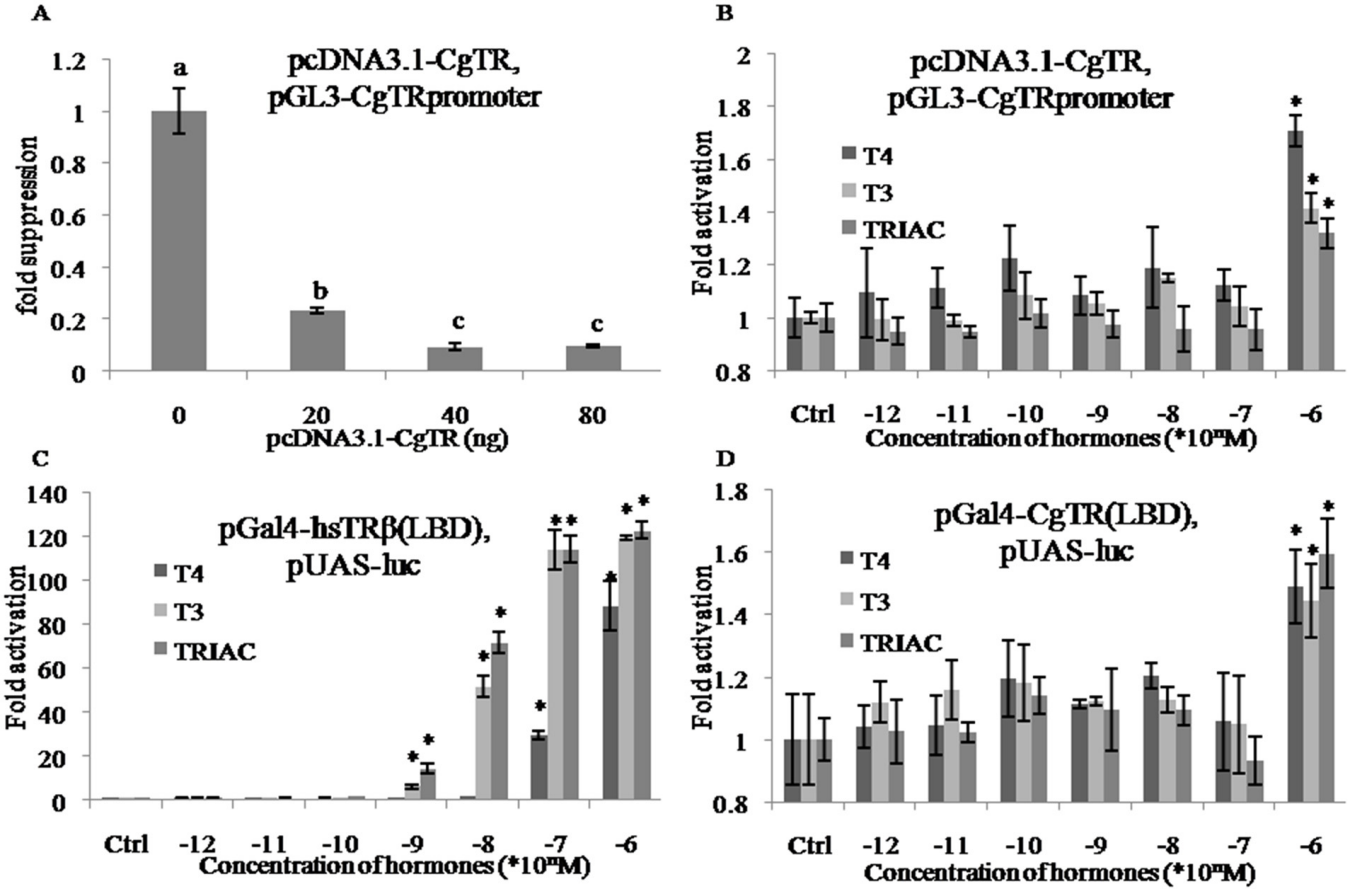
Transcriptional activity of *CgTR* could not be activated by T4, T3 and TRIAC. (A) Transcriptional repression of the *CgTR* promoter by increasing amount of pcDNA3.1-CgTR (0, 20, 40, 80 ng) in absence of THs. Multiple comparisons were analyzed by one-way ANOVA in SPSS. (B) The transcriptional regulation activity of CgTR protein was hardly activated by increasing concentration of T4, T3 and TRIAC. (C) The transcriptional regulation activity of hsTRβ LBD was activated by increasing concentration of T4, T3 and TRIAC. (D) The transcriptional regulation activity of CgTR LBD was hardly activated by increasing concentration of T4, T3 and TRIAC. Data are represented as the mean ± SD of triplicate independent experiments. In (B), (C) and (D), data were pairwise compared to the corresponding control by Student’s *t* test and statistically significant difference are indicated by asterisks (*p < 0.05).

To investigate whether the expression of *CgTR* could be regulated by CgTR protein, dual-luciferase reporter assays using pcDNA3.1/pGL3 were performed in HEK293T cells in the absence of THs. Co-transfection of increasing amounts of pcDNA3.1-CgTR (0, 20, 40, or 80 ng) with the pGL3-CgTRpromoter reporter gene in the absence of THs resulted in transcriptional repression of the *CgTR* promoter in a dose-dependent manner (Fig 7A). This result is consistent with the results reported for *X*. *laevis* that xlTR protein repressed the transcription activity of *xlTRβA* promoter in the absence of THs [27]. This indicates that CgTR can directly inhibit the expression of *CgTR* mRNA and also supports the transcriptional repression activity of CgTR.

To further investigate the effect of ligand-coupled CgTR on the transcriptional activation of *CgTR* mRNA, the amount of plasmid pcDNA3.1-CgTR was fixed at 20 ng/well and different concentrations of T4, T3, or TRIAC were added in an attempt to activate the transcriptional activity of CgTR. The results indicated that both THs and a TH derivative had no effect on the transcriptional activity of CgTR, except at the highest concentration (10^−6^M). When 10^−6^M T4, T3, or TRIAC was added, only an approximate 1.5-fold activation was observed (Fig 7B).

To confirm this result, a second dual-luciferase reporter system using pGal4/pUAS was adopted. In this system, Gal4-DBD was linked in front of CgTR LBD and the Gal4-responsive luciferase reporter pUAS-luc was used as a reporter plasmid. This system excluded the influence of CgTR DBD and examined the transcriptional activity of LBD alone. In the reporter system using pGal4-hsTRβ(LBD), which served as positive control, the highest activation of the reporter gene by T3 or TRIAC was approximately 120-fold higher than that of the control, whereas for T4, the activation was 88-fold higher (Fig 7C). When pGal4-CgTR(LBD) was used, T4, T3, or TRIAC still failed to initiate the transcriptional activity of CgTR LBD. As in the dual-luciferase reporter assays using pcDNA3.1/pGL3, only an approximate 1.5-fold activation was observed with 10^−6^M T4, T3, or TRIAC (Fig 7D). Given the low activation effects and high concentration of hormones required, T4, T3, and TRIAC could not trigger the transcriptional activity of CgTR at relevant physiological concentrations *in vitro*.

## Discussion

Although TH functions have for decades been regarded as a synapomorphy of vertebrates, recent studies have dated the origin of TH functions back to the chordates. On the basis of the sporadic evidence previously reported, we hypothesized that THs are functional in non-chordate invertebrates, and accordingly, we selected *C*. *gigas* as a model to study TH-related functions and their molecular mechanisms in oyster development. THs were first qualitatively and quantitatively detected in *C*. *gigas*. Then, by searching the Pacific oyster genome [28], we found orthologs of critical genes in the TH signaling pathway, including several thyroid peroxidases, two deiodinases, one RXR, and one TR. Among these genes, two deiodinases were identified and reported in our previous work [29]; the single TR and RXR were found in the Pacific oyster genome using bioinformatics methods [26]. Taken together, these results suggest that there is an active TH signaling system in oysters. As the first step in verifying this hypothesis, this study focused on the presence of THs and the functional characterization of TR in *C*. *gigas*.

### THs are present in oysters and may be involved in embryogenesis and metamorphosis

To date, THs have been detected in other mollusks using ELISA and thin-layer chromatography (TLC) in the scallop *C*. *farreri* [14] and in the sea hare *Aplysia californica* [12], respectively. However, antibodies may cross-link with similar substrates when using the ELISA method and TLC has the disadvantage of low sensitivity and resolution ratio. The two methods therefore did not provide direct evidence in qualitative analysis. Here, we used two sensitive and reliable methods (HPLC and LC/MS) for qualitative measurement of oyster THs. Our results further confirm the presence of THs in mollusks and provide the foundation for further quantitative measurement of THs using antibody-based methods (i.e., the electrochemical immuno-assay). In the TH quantitative experiments, due to the disturbance of the shells formed begin with D-shape larva and the enormous number of larvae used in each sample, it is hard to normalize TH contents with total weight or larva number. Here, protein contents in freeze-dried samples of were determined (showed in S1 Table) and used to normalize TH contents in different developmental stages. The quantitative results (Fig 2A and 2B) not only further confirm the presence of THs in all examined developmental stages but also provide evidence for their possible physiological function.

Variation in the T3 and T4 contents remained consistent during the early developmental stages, and the TH contents increased continually during embryogenesis and peaked at the gastrula stage. This result supports the conclusion that THs may be involved in oyster embryogenesis. THs are also important in vertebrate embryogenesis [30] and a similar TH variation tendency during embryogenesis of the fathead minnow, *Pimephales promelas*, has been reported [31]. It is well known that TH-mediated metamorphosis is an ancestral feature of chordates [32]. Interestingly, metamorphosis of two mollusks, *Haliotis discus* and *H*. *gigantea*, was also reported to be induced by T4 and T3 [13]. Our data showed that the level of T3 was significantly higher at 1.5 dps and 4 dps than in the pre-metamorphosis stage pediveliger, which is regarded as the metamorphosis-competent stage. The larvae at 1.5 dps have already completed settlement and initiated metamorphosis. Thus, THs are likely to be involved in the metamorphosis process of oysters.

### Oysters may synthesize THs endogenously

A homolog of the vertebrate thyroid gland has yet to be identified in oysters. This raises the question about the sources of THs in oyster larvae. The possible sources include maternal inheritance, food supply, and autonomous biosynthesis. Maternal-derived THs have been detected in unfertilized oyster eggs (Fig 2A and 2B). These THs have been reported to be essential in the embryonic development of vertebrates before the embryonic thyroid gland commences hormone secretion [30,33]. Moreover, T4 and T3 were detected in four species of algae that oyster larvae feed on (S4 Fig), which suggests that at least some of the oyster THs may be derived from these algae consumed during the feeding period (from D2 to 4 dps). This is also consistent with the opinion of Heyland *et al*. [34] that some of the T4 in sea urchin larvae was derived from feeding on algae. Most importantly, the TH contents increased more than 2-fold during the non-feeding embryogenesis stages, from the egg to the gastrula stage, suggesting that oyster embryos may synthesize THs endogenously (Fig 2A and 2B). Furthermore, a thyroid peroxidase homolog (named PERT1) is expressed mainly in the blastula (B), gastrula (G), and trochophore (T) stages (S5 Fig). The expression pattern of PERT1 fits the putative THs synthesis stages well, which further supported the THs synthesis assumption. Meanwhile, PERT1 may be the main effective gene in the THs synthesis. Correspondingly, a TLC assay indicated that the mollusk *A*. *californica* can use incorporated I^125^ to synthesize THs and that this TH synthesis can be blocked by thiourea [12]. This evidence suggests that mollusks can synthesize THs endogenously. However, to date, no homolog of the vertebrate thyroid gland has been found in mollusks. Further studies that focus on how and where THs are synthesized in oysters will be helpful in understanding the evolution of TH biosynthesis.

### The conserved function of *CgTR*

In chordates, the biological activities of THs are mediated by TRs through the transcriptional regulation of TH-responsive genes [27]. To investigate the molecular mechanism of the TH signaling cascade, we cloned the single TR gene (*CgTR*) and further studied its functional characteristics. The phylogenetic relationship of CgTR and other known TRs was figured out by phylogenetic analysis. The result showed that the jawless vertebrate TRs were separated from the jawed vertebrate TRs. Escriva et al. argued that vertebrate TRs underwent duplication after the split of jawless and jawed vertebrates. This scenario was consistent with their hypothesis that the second round whole genome duplication arose after the split of jawless and jawed vertebrates [35]. However, increasing evidence supports another hypothesis that the first round and the second round whole genome duplication occurred before the split of jawless and jawed vertebrates [36,37]. If this hypothesis is true, this topology may be an artifact produced by long-branch attraction if the two genes (pmTR1 and pmTR2) evolved rapidly [36]. Another possibility is that the ancestral TR may had duplicated twice before the split of jawless and jawed vertebrates, which was then followed by the independent loss of the two highly relevant TRs in jawless and jawed vertebrates. Like other chordate TRs, CgTR is also localized in the cell nucleus (S6 Fig), which provides the spatial basis for transcriptional regulation. Furthermore, the dual-luciferase reporter system with pcDNA3.1/pGL3 indicated that CgTR has transcriptional repression activity (Fig 7A). The yeast two-hybrid assay (Fig 5) suggested that CgTR could form a heterodimer with CgRXR. This is the first evidence of an interaction between TR and RXR in mollusks. There is other evidence in Lophotrochozoa that the platyhelminth *S*. *mansoni* TR (SmTR) can also interact with SmRXR1, which is supported by GST pull-down experiments [15]. Thus, we conclude that the ability to form a heterodimer with RXR has occurred in the common ancestor of protostome and deuterostome TRs.

The *X*. *laevis* TRβA promoter, which is a well-characterized direct T3-responsive promoter, contains a *bona fide* TRE [38]. TR expression can be induced by THs in vertebrates and amphioxus, and the subsequent autoinduction is an important transcriptional event in the biological action of THs [4,9]. In this study, CgTR protein expression was correlated with THs variation during embryogenesis and *CgTR* mRNA was significantly induced by exogenous T4 *in vivo* (Figs 2 and 4). Thus, CgTR may be involved in TH signaling. The expression of CgTR was induced within 1 h, which seems too fast to be usual genomic actions. In *Xenopus laevis*, the amount of TR transcripts increased 4–6 h post-treatment [39]. This difference, however, may be due to different developmental stages and different organisms used in the two experiments. Moreover, a putative atypical TRE (CgDR5) was found in the promoter of *CgTR*, and EMSA experiments also confirmed the binding of CgTR to CgDR5 (Fig 7B). Results from the dual-luciferase reporter system with pcDNA3.1/pGL3 demonstrated that over expression of the unliganded CgTR caused a substantial repression of basal transcriptional activity of the *CgTR* promoter (Fig 7A), similar to vertebrate TRs [27]. Thus, the expression of *CgTR* mRNA may be repressed by CgTR protein via binding to the TRE in the promoter of *CgTR*. These results demonstrate that some CgTR functions are conserved, suggesting that these TR functions had developed in the common ancestor of protostome and deuterostome TRs.

### The DNA binding specificity and transcriptional activity of *CgTR* differ from those of vertebrate TRs

The EMSA results suggest that CgTR can repress gene expression through binding to the promoters of target genes, as in vertebrates. However, the DNA-binding specificity of CgTR is not identical to that of vertebrate TRs, which specifically bind to DR0 and DR4 [6]. The EMSA experiments in this study showed that CgTR can bind to DR0-DR5. Similar EMSA results were observed in the platyhelminth *S*. *mansoni* with SmTRs as the target protein [15]. The binding specificity of nuclear receptors is determined by the DBD domain. The P-box is the most important short protein motif determining the target DNA sequence [40]. The P-box of CgTR and SmTRs is identical with that of vertebrate TRs, which may dictate that these TRs bind the specific response element AGGTCA. The DR-box is important in determining the spacing between the direct repeats [40]. The DR-box of CgTR has two residues changed compared to that of vertebrate TRs (S1 Fig), which may determine the binding of CgTR to DR0-DR5. TRs have gained the ability to bind the direct repeats of the consensus half-site in the common ancestor of protostome and deuterostome TRs. However, Lophotrochozoa TRs did not subsequently evolve a distinct binding specificity in regard to the spacing between half-sites, as described in vertebrate TRs.

The results from the two dual-luciferase reporter assays using either pcDNA3.1/pGL3 or pGal4/pUAS indicated that T4, T3, and TRIAC could not initiate the transcriptional activity of CgTR at relevant physiological concentrations *in vitro*. In the two dual-luciferase reporter systems, activation of luciferase expression needs two indispensable conditions: first that the ligand-coupled CgTR has a genuine transcriptional activity and that CgTR binds to T4, T3, or TRIAC. Thus, there are two possible reasons to explain the negative experimental results. Foremost, CgTR did not have transcriptional activity. The divergence of AF2-AD motif may have disabled the transcriptional activity of CgTR. The amino acids in AF2-AD motif, which has a core structure of ΦΦXEΦΦ, where Φ denotes a hydrophobic residue, play an important role in recruiting a coactivator [41]. The vertebrate TRs have an identical AF2-AD of LFLE(V/I)F, whereas CgTR has a less conserved AF2-AD motif with a cores structure of QLLDLF, in which four out of six residues have been substituted with a hydrophilic Q residue in the first position (S1 Fig). In this context, CgTR may not mediate TH signaling and THs may conduct physiological effects through other signaling pathways in oysters. Actually, another mollusk nuclear receptor, the *Octopus vulgaris* estrogen receptor, which does not bind estradiol and is unresponsive to estrogens, may also not mediate estrogen signaling [42].

The other possible reason is that CgTR may not bind to T4, T3, and TRIAC. In this hypothesis, it seems contradictory that T4 and T3 are detected throughout oyster developmental stages and oyster embryos may synthesize THs endogenously (Figs 1 and 2). Furthermore, the T4 and T3 treatment on oyster larvae can induce the expression level of CgTR, and metamorphosis of two mollusks was reported to be induced by T4 and T3 [13]. A possbile explanation for this paradox may be that the ligand of CgTR is another TH derivative and that T4 and T3, analogous to T4 in vertebrates, may be the prohormones. Indeed, the TR ligand has changed over the course of evolution. Vertebrate TRs mainly bind to T3, whereas the amphioxus TR binds to TRIAC rather than to T4 or T3. However, T4 and T3 still could induce the metamorphosis of amphioxus [9]. T4 and T3 may be transformed to the active form TRIAC *in vivo* and subsequently affect the metamorphosis of amphioxus. Active metabolism of thyroid hormone was also demonstrated in amphioxus [43,44]. By analyzing the amino acid sequences of TR LBD, we found that the 18 residues that make ligand contact were uniformly conserved across vertebrate species. Most of the ligand selectivity in binding derives from these critical residues [45]. However, nine and eight of these residues changed in CgTR and amphioxus TR, respectively. The amphioxus TR binds to TRIAC rather than to T4 or T3, whereas CgTR may not bind to T4, T3, or TRIAC. In contrast to the amphioxus TH system and TR, it seems reasonable to speculate that the ligand of CgTR is other TH derivatives; Indeed, 3,3′-diidothyronine, diiodotyrosine, and monoiodotyrosine were identified by paper chromatography in snails [46, 47]. These TH derivatives may be the possible ligands of CgTR and need to be tested in the following-up experiments. However, it is also possible that CgTR could not bind any THs or derivatives.

## Conclusions

In this study, T4 and T3 were qualitatively detected by HPLC and LC/MS in *C*. *gigas* and quantitatively measured throughout the developmental stages. The quantitative results suggest that THs may be involved in the embryogenesis of oysters and that oyster embryos may synthesize THs endogenously.

The single CgTR was cloned and its functional characteristics were studied. The results demonstrate that some CgTR functions are conserved. However, the DNA binding specificity of CgTR is different from that of vertebrate TRs, which could bind to DR0-DR5. Experiments with two dual-luciferase reporter systems indicated that CgTR is unresponsive to T4, T3, and TRIAC. Our experimental results will provide important evidence in understanding the evolution of TR function and the TH system.

## Supporting Information

S1 Fig Multiple alignment of the *CgTR* with other known TRs. A, alignment of the DNA binding domain (DBD) of TRs. A highly conserved motif named the 'N-terminal signature sequence' (NTSS) is found at the 3' end of A/B domain. Stars indicate the conserved cysteine residues that comprise the zinc finger of the DBD. The conserved DR-box, P-box, T-box, and A-box are figured out. B, alignment of the ligand binding domain (LBD) of TRs. Helices (H3-H12), Motif I, Motif II and autonomous activation domain (AF2-AD) are indicated. Nine out of eighteen residues that make ligand contacts, denoted by a dark sphere, are not conserved in CgTR. hs, *Homo sapiens* (hsTRa, AB307686; hsTRb, M26747); rn, *Rattus norvegicus* (rnTRa, M18028; rnTRb, J03933); xl, *Xenopus laevis* (xlTRa, M35343; xlTRb, M35360); ol, *Oryzias latipes* (olTRa, AB114860; olTRb, AB114861); pm, *Petromyzon marinus* (pmTR1, DQ320317; pmTR2, DQ320318); bl, *Branchiostoma lanceolatum* (blTR, EF672345); sm, *Schistosoma mansoni* (smTRa, AY395038; smTRb, AY395039); sj, *Schistosoma japonicum* (sjTRb, JX111998); cg, *Crassostrea gigas* (cgTR, KP271450).
(TIF)

S2 Fig Schematic representation of the genomic structure of *CgTR*. A putative atypical TRE (CgDR5) was found in the *CgTR* promoter at -1043 to -1070, with five base pairs were inserted between the direct repeats and two mismatches in the first half-site, compared with the consensus AGGTCA.
(TIF)

S3 Fig Recombinant expression and western blot analysis of CgTR. Lane 1 and 2, SDS-PAGE analysis of un-induced and IPTG induced plasmid transfected expression bacteria, respectively. Lane 3 and 4, western blot analysis of rCgTR contained bacteria protein and total protein of trochophore larvae using anti-CgTR antibody. Arrow indicated the band of CgTR and star indicated the non-specific band.
(TIF)

S4 Fig Four algae species commonly used as larval nutrition in oyster larvae cultures contain T3 and T4. Four algae species *Isochrysis galbana* (I. gal), *Nitzschia closterium f*. *minutissima* (N. cfm), *Chlorella salina* (C. sal), *Tetraselmis chui* (T. chu) were collected and vacuum freeze dried. THs extraction and quantitative measurement was conducted as that in oyster larvae. Data were standardized by dry weight and presented as mean ± SD of triplicate independent experiments.
(TIF)

S5 Fig Expression patterns of the thyroid peroxidase homolog (PERT1) transcripts in the developmental stages, determined by quantitative real-time PCR. *RS18* gene expression was used as an internal control. Data are normalized to the egg stage (E) and displayed as the mean ± SD of triplicate independent experiments.
(TIF)

S6 Fig Subcellular localization of *CgTR* fused to EGFP. Fluorescence images of Hela cells, transient transfection of control pEGFP-N1 (A-C) and pCgTR-EGFP (D-F). Images A, D showed DAPI stained nuclei, images B, E showed EGFP expression, image C, F are merged images of A-B and D-E. White bars represent 10 μm.
(TIF)

S1 Table Protein contents of freeze-dried samples in different developmental stages. E: egg, B: blastula, G: gastrula, T: trochophore, D: D-shape, U: umbo, P: pediveliger, dps, days post-settlement.
(TIF)
