# Supplementary figures and images for: Identification of Thyroid Hormones and Functional Characterization of Thyroid Hormone Receptor in the Pacific Oyster Crassostrea gigas Provide Insight into Evolution of the Thyroid Hormone System
Source: PLoS One. 2015 Dec 28;10(12):e0144991. doi: 10.1371/journal.pone.0144991 (PMC4692385; doi:10.1371/journal.pone.0144991)

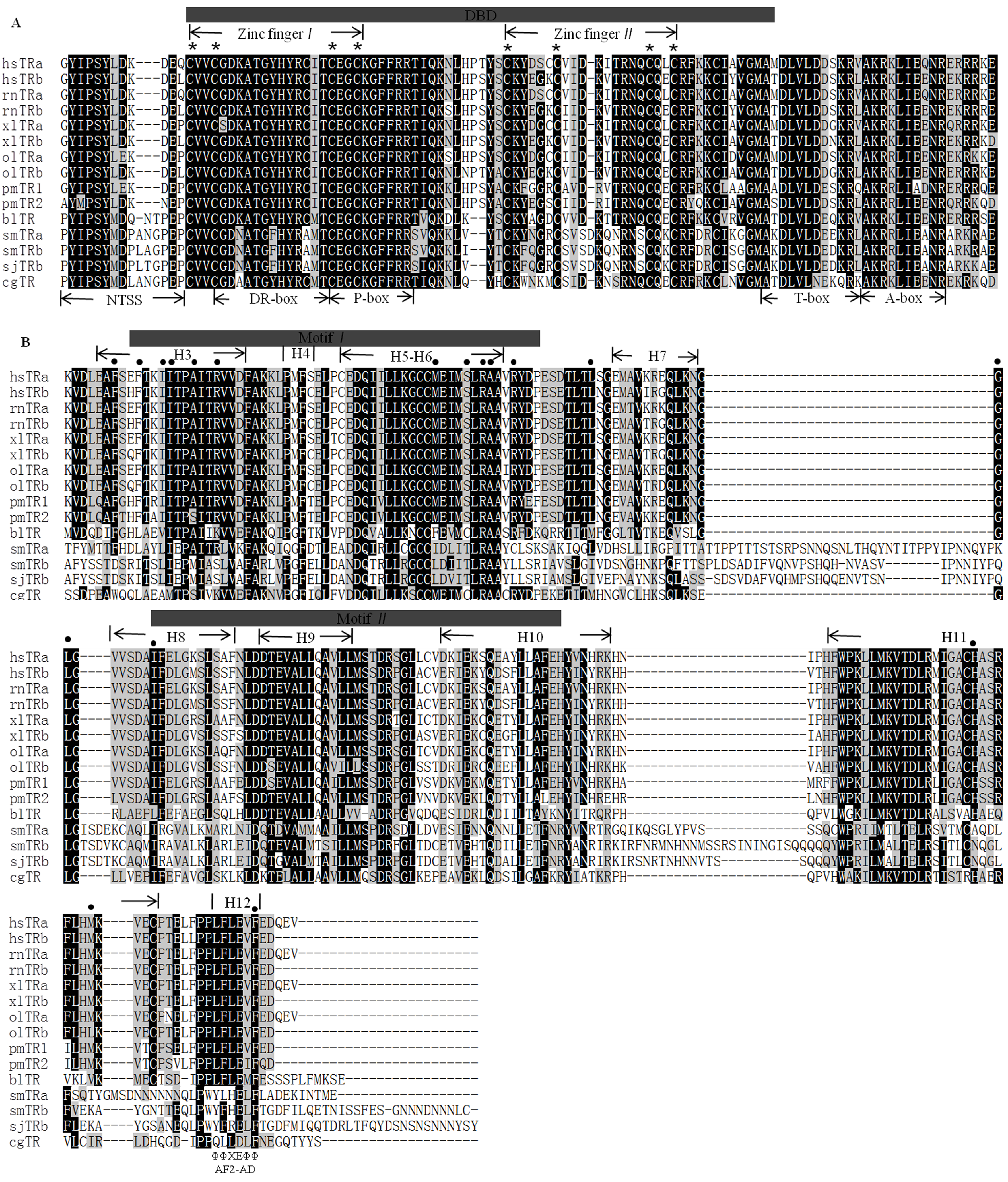

Supplement: S1 Fig — A, alignment of the DNA binding domain (DBD) of TRs. A highly conserved motif named the 'N-terminal signature sequence' (NTSS) is found at the 3' end of A/B domain. Stars indicate the conserved cysteine residues that comprise the zinc finger of the DBD. The conserved DR-box, P-box, T-box, and A-box are figured out. B, alignment of the ligand binding domain (LBD) of TRs. Helices (H3-H12), Motif I, Motif II and autonomous activation domain (AF2-AD) are indicated. Nine out of eighteen residues that make ligand contacts, denoted by a dark sphere, are not conserved in CgTR. hs, Homo sapiens (hsTRa, AB307686; hsTRb, M26747); rn, Rattus norvegicus (rnTRa, M18028; rnTRb, J03933); xl, Xenopus laevis (xlTRa, M35343; xlTRb, M35360); ol, Oryzias latipes (olTRa, AB114860; olTRb, AB114861); pm, Petromyzon marinus (pmTR1, DQ320317; pmTR2, DQ320318); bl, Branchiostoma lanceolatum (blTR, EF672345); sm, Schistosoma mansoni (smTRa, AY395038; smTRb, AY395039); sj, Schistosoma japonicum (sjTRb, JX111998); cg, Crassostrea gigas (cgTR, KP271450). (TIF) [file pone.0144991.s001.tif]

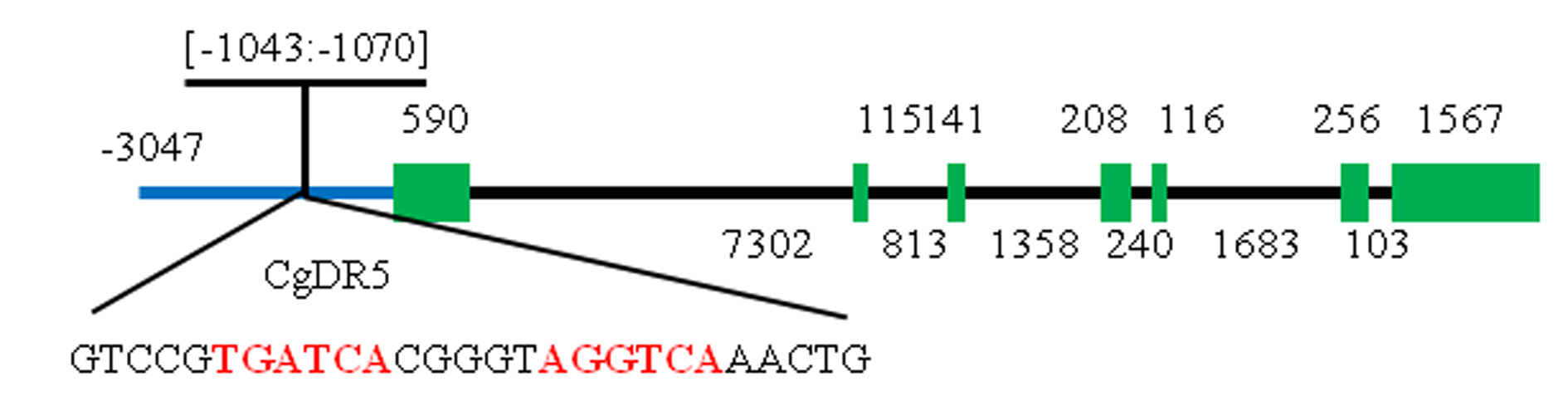

Supplement: S2 Fig — A putative atypical TRE (CgDR5) was found in the CgTR promoter at -1043 to -1070, with five base pairs were inserted between the direct repeats and two mismatches in the first half-site, compared with the consensus AGGTCA. (TIF) [file pone.0144991.s002.tif]

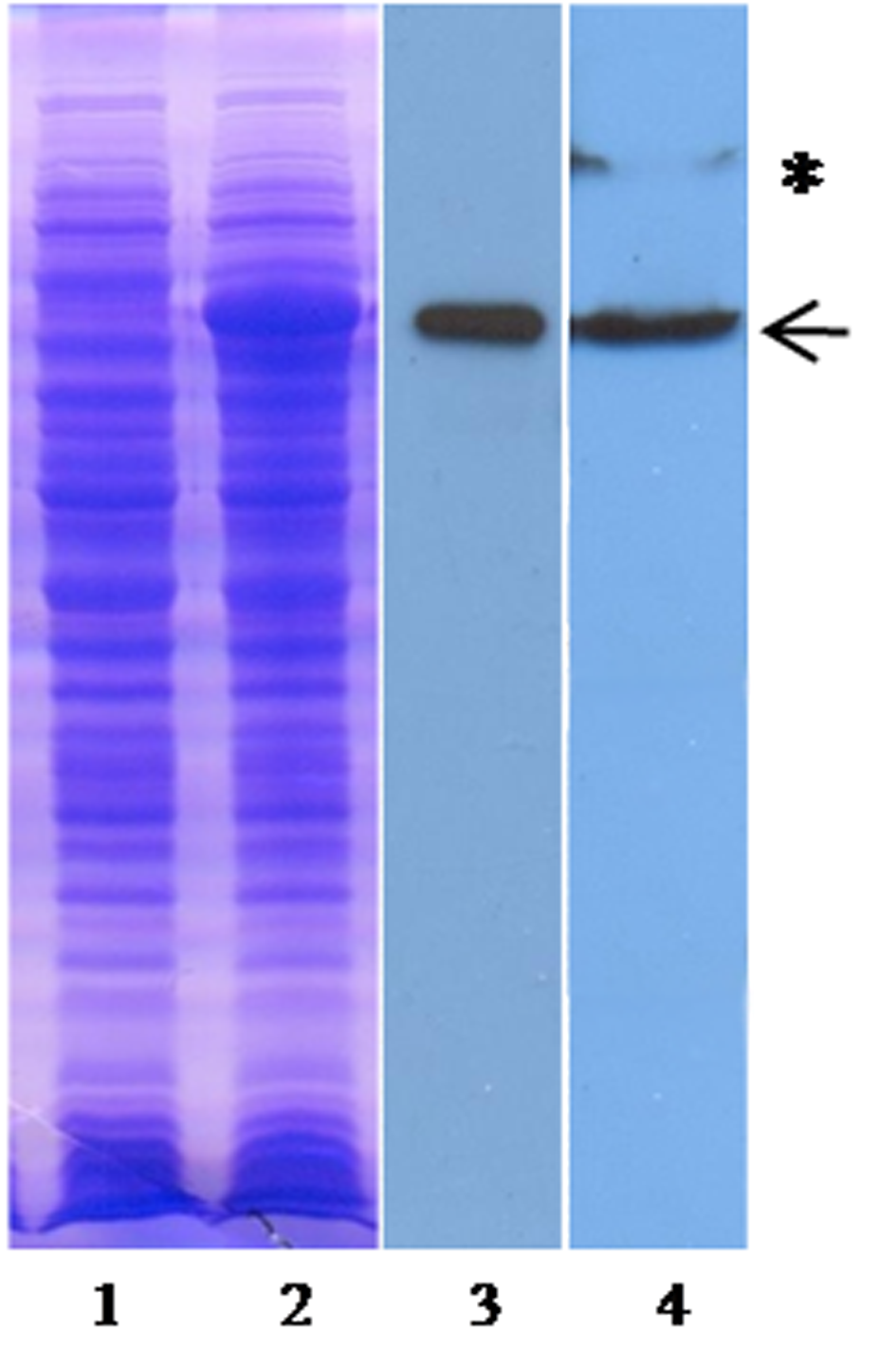

Supplement: S3 Fig — Lane 1 and 2, SDS-PAGE analysis of un-induced and IPTG induced plasmid transfected expression bacteria, respectively. Lane 3 and 4, western blot analysis of rCgTR contained bacteria protein and total protein of trochophore larvae using anti-CgTR antibody. Arrow indicated the band of CgTR and star indicated the non-specific band. (TIF) [file pone.0144991.s003.tif]

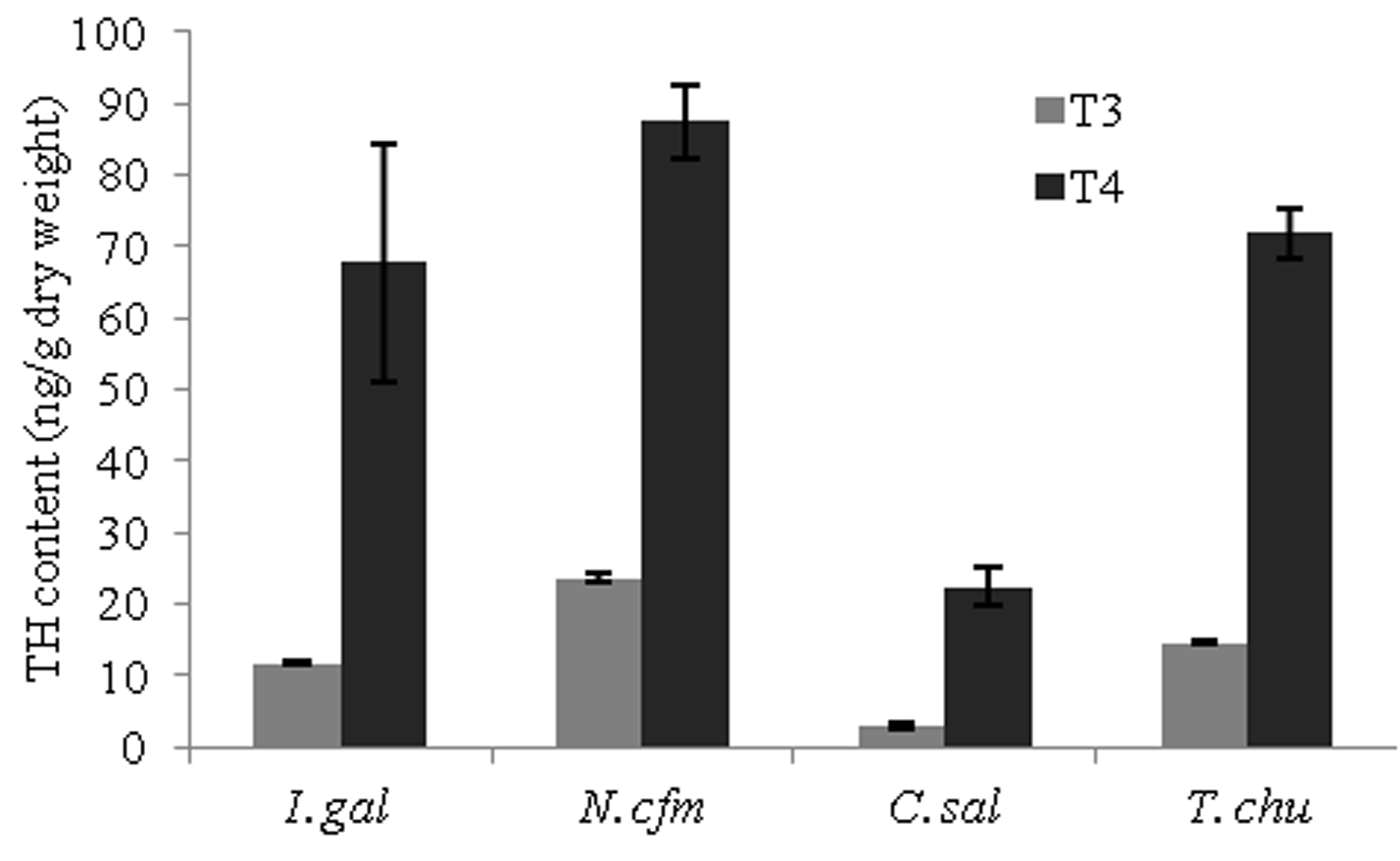

Supplement: S4 Fig — Four algae species Isochrysis galbana (I. gal), Nitzschia closterium f. minutissima (N. cfm), Chlorella salina (C. sal), Tetraselmis chui (T. chu) were collected and vacuum freeze dried. THs extraction and quantitative measurement was conducted as that in oyster larvae. Data were standardized by dry weight and presented as mean ± SD of triplicate independent experiments. (TIF) [file pone.0144991.s004.tif]

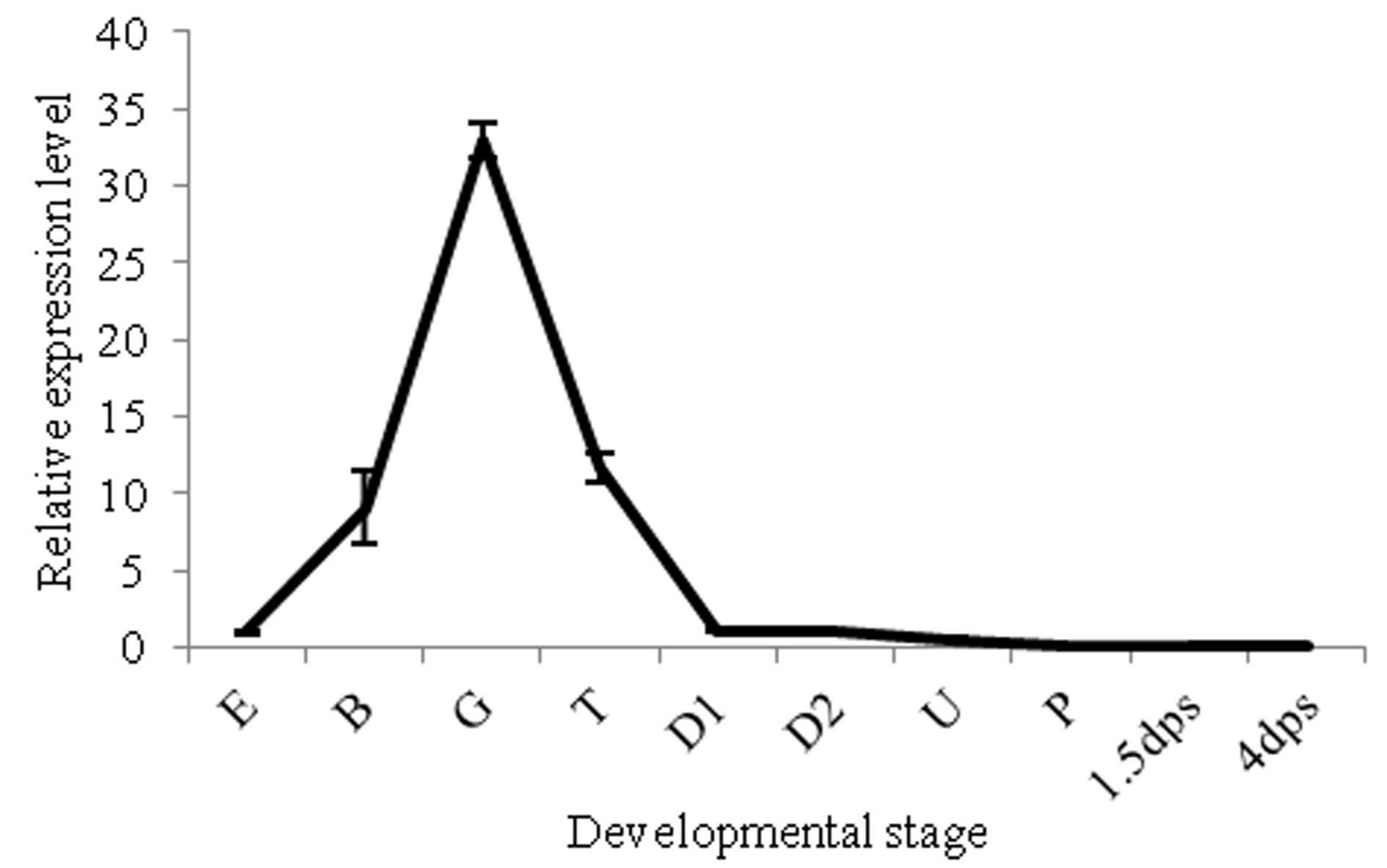

Supplement: S5 Fig — RS18 gene expression was used as an internal control. Data are normalized to the egg stage (E) and displayed as the mean ± SD of triplicate independent experiments. (TIF) [file pone.0144991.s005.tif]

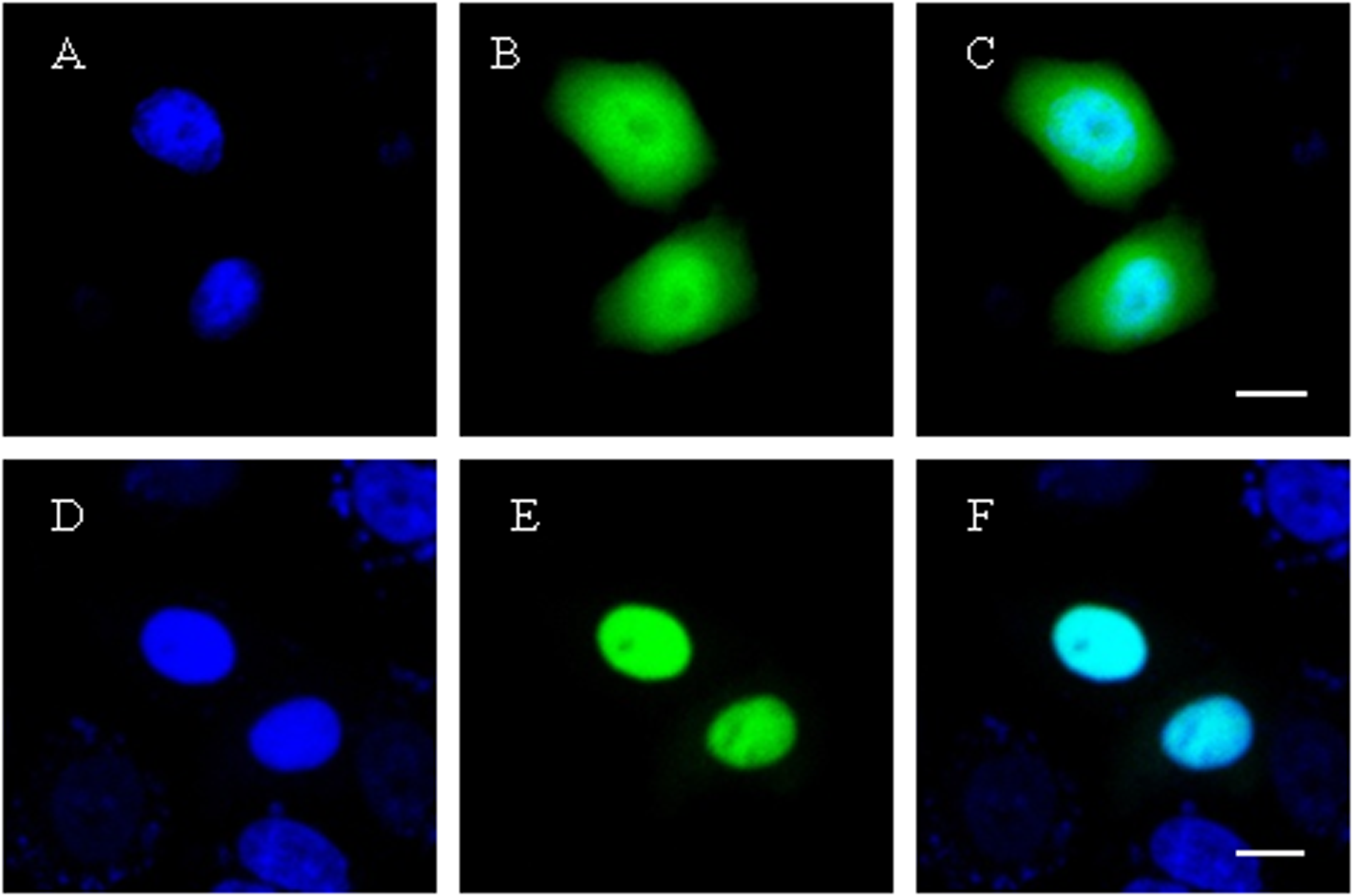

Supplement: S6 Fig — Fluorescence images of Hela cells, transient transfection of control pEGFP-N1 (A-C) and pCgTR-EGFP (D-F). Images A, D showed DAPI stained nuclei, images B, E showed EGFP expression, image C, F are merged images of A-B and D-E. White bars represent 10 μm. (TIF) [file pone.0144991.s006.tif]

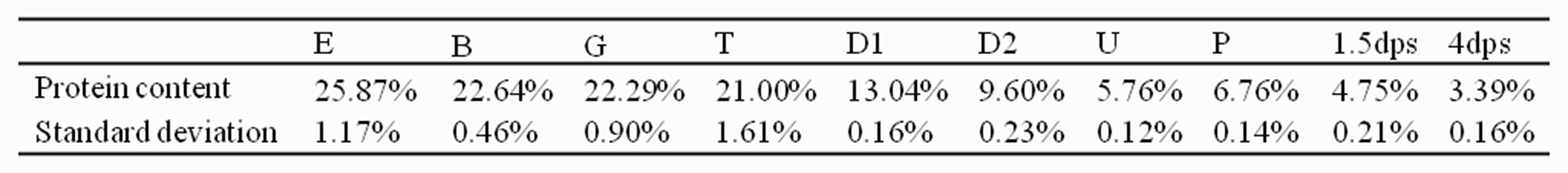

Supplement: S1 Table — E: egg, B: blastula, G: gastrula, T: trochophore, D: D-shape, U: umbo, P: pediveliger, dps, days post-settlement. (TIF) [file pone.0144991.s007.tif]
